# Gender Bias in Diagnosis, Prevention, and Treatment of Cardiovascular Diseases: A Systematic Review

**DOI:** 10.7759/cureus.54264

**Published:** 2024-02-15

**Authors:** Abdullah Al Hamid, Rachel Beckett, Megan Wilson, Zahra Jalal, Ejaz Cheema, Dhiya Al-Jumeily OBE, Thomas Coombs, Komang Ralebitso-Senior, Sulaf Assi

**Affiliations:** 1 Pharmacy Practice, King Faisal University, Al Ahsa, SAU; 2 Forensic Science, Liverpool John Moores University, Liverpool, GBR; 3 Pharmacology and Therapeutics, Birmingham University, Birmingham, GBR; 4 Pharmacy, University of Management and Technology, Lahore, PAK; 5 Computer Science and Mathematics, Liverpool John Moores University, Liverpool, GBR; 6 Toxicology, British American Tobacco, Southampton, GBR; 7 Pharmacy, Liverpool John Moores University, Liverpool, GBR

**Keywords:** women’s health, cardiovascular disease prevention, diagnosis and treatment, cardiovascular diseases (cvd), gender bias

## Abstract

Cardiovascular disease (CVDs) has been perceived as a ‘man’s disease’, and this impacted women’s referral to CVD diagnosis and treatment. This study systematically reviewed the evidence regarding gender bias in the diagnosis, prevention, and treatment of CVDs. Preferred Reporting Items for Systematic Reviews and Meta-analyses (PRISMA) guidelines were followed. We searched CINAHL, PubMed, Medline, Web of Science, British Nursing Index, Scopus, and Google Scholar. The included studies were assessed for quality using risk bias tools. Data extracted from the included studies were exported into Statistical Product and Service Solutions (SPSS, v26; IBM SPSS Statistics for Windows, Armonk, NY), where descriptive statistics were applied. A total of 19 studies were analysed. CVDs were less reported among women who either showed milder symptoms than men or had their symptoms misdiagnosed as gastrointestinal or anxiety-related symptoms. Hence, women had their risk factors under-considered by physicians (especially by male physicians). Subsequently, women were offered fewer diagnostic tests, such as coronary angiography, ergometry, electrocardiogram (ECG), and cardiac enzymes, and were referred to less to cardiologists and/or hospitalisation. Furthermore, if hospitalised, women were less likely to receive a coronary intervention. Similarly, women were prescribed cardiovascular medicines than men, with the exception of antihypertensive and anti-anginal medicines. When it comes to the perception of CVD, women considered themselves at lower risk of CVDs than men. This systematic review showed that women were offered fewer diagnostic tests for CVDs and medicines than men and that in turn influenced their disease outcomes. This could be attributed to the inadequate knowledge regarding the differences in manifestations among both genders.

## Introduction and background

Cardiovascular diseases (CVDs) represent the leading cause of mortality worldwide accounting for 17.9 million deaths each year [1]. CVDs refer to several disorders that affect the heart and blood vessels including coronary heart disease (CHD) also known as ischaemic heart disease (IHD), strokes and transient ischaemic attack (TIA or mini stroke), peripheral arterial disease (PAD) and aortic disease [2].

CVDs have been commonly perceived as ‘men’s disease’ and this misconception has contributed to under-diagnosis and treatment for women worldwide [3]. In comparison to men, women are 50% more likely to be misdiagnosed with a heart attack even though they carry the same risk of developing CVDs as men [4]. It has been known for over two decades that women experience CVDs differently to men [5,6]; yet both genders are still considered the same by healthcare professionals despite the presence of gender-specific requirements in many guidelines [7-13]. Moreover, mortality linked to CVDs is higher globally in women [14].

Prevention of CVDs is mainly associated with modifying the risk factors including body mass index (BMI), smoking, high blood pressure, high blood cholesterol, lack of physical activity and diabetes [4]. Some of these risk factors such as smoking and obesity have been shown to have a greater impact on women than men [15,16]. The risk of CVDs is more exponential in women with a sudden increase in risk once they reach around 60 years of age [17,18]. 

Diagnosis of CVDs is not straightforward in women due to the different and delayed presentation of symptoms when compared to men. Women with acute myocardial infarction (MI) are reported to present with atypical symptoms including abdominal pain, dyspnoea, nausea, back and neck pain, indigestion, palpitations and unexplained fatigue; as opposed to a well-defined chest pain, which is the typical men presentation and often better recognised by doctors [19,20]. Furthermore, women are less likely to report their symptoms and know the risks of CVDs [21]. Lack of symptom reporting in turn lead to delayed/under-diagnosis and subsequent treatment and that negatively affect the clinical outcomes [17,22]. The misconception that CVDs represent men’s diseases has led women to believe that they are at a lower risk of CVDs.

The current prevention, diagnosis and treatment approaches of CVDs adopted by physicians failed to consider the two genders (men and women) as physiologically different. These biases have therefore contributed to the lack of awareness of CVDs risk in women contributing to their delayed diagnosis and poor clinical outcomes. Furthermore, the different manifestation of CVDs in men and women together with the limited representation of women in the existing evidence suggest that the current evidence is largely representative of the CVDs in men. This systematic review, therefore, aimed to examine the current evidence related to genderbias in the diagnosis, prevention and treatment of CVDs. 

## Review

Methods

Search Strategy

We based our study on the Preferred Reporting Items for Systematic Reviews and Meta-analyses (PRISMA) (Appendix, Supplementary file S1) [23-60]. We searched CINAHL, Medline, PubMed, Web of Science, British Nursing Index, Scopus, and Google Scholar by using a combination of MeSH subject headings, keywords and titles, abstract, and full-text keywords, with publication dates between 2000 and 2021. The primary search terms used through the aforementioned databases were ‘Cardiovascular disease’ and ‘Women’. The terms 'Gender bias', 'Prevention', 'Diagnosis', and 'Treatment', and their synonyms were later added to obtain more relevant studies. The search strategy for Medline is specified in Supplementary file S2 (Appendix). No language or publication status limits were applied to the search strategy.

Inclusion and Exclusion Criteria

**Figure 1:**
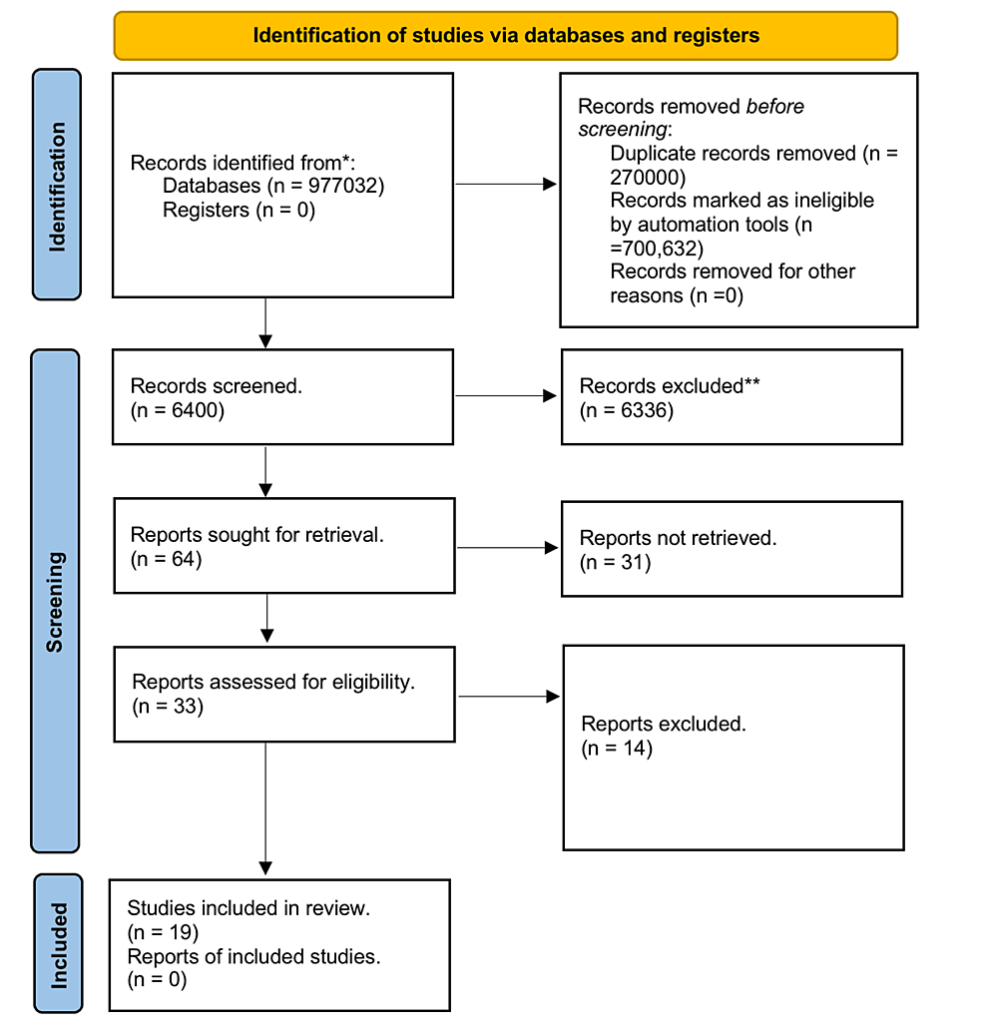
Extraction of studies considering the PRISMA guidelines (Supplementary files S1 and S2)

Studies eligible for inclusion were peer-reviewed articles published between January 2000 and December 2021. We included studies that investigated CVDs among adult women. We excluded studies that did not specify the women-to-men ratio, conference abstracts, and randomised controlled trials where women are often under-represented [24]. Moreover, we excluded studies that investigated mainly other conditions than CVDs, such as diabetes and biochemical and/or molecular mechanisms. The title of the study was initially assessed according to the inclusion/exclusion criteria, followed by the abstract and then full text. Figure 1 shows the flow chart regarding the study selection.

Data Extraction and Quality Assessment

Data were extracted by Rachel Beckett (RB) and reviewed by Sulaf Assi (SA) for accuracy and completeness. Any disagreement was resolved by discussion. The extracted studies were organised by study type, and the following characteristics were included for each study: study settings, participants’ characteristics, sample size, men-to-women ratio, main diagnosis, treatment, risk factors, and awareness of patients regarding CVDs’ preventive measures [33-52] (Table 1).

**Table 1:** Characteristics of the studies included in the review Occurrence of all CV events referred to at least one event of CV death, myocardial infarction, hospitalisation for unstable angina or HF, cerebrovascular accident, or emergency revascularisation. ACS: acute coronary syndrome, AMI: acute myocardial infarction, CABG/AV: coronary artery bypass graft surgery/atrioventricular, CAD: coronary artery disease, CT: computed tomography, CVD: cardiovascular diseases, CHD: congestive heart disease, HF: heart failure, MI: myocardial infarction, PAD: peripheral artery disease, PCI: percutaneous coronary intervention

Study/Type			Country	Setting	Patient Characteristics	Main Diagnosis	Main outcome measure	Duration	Sample Size	M:W
		Cohort								
Clerc Liaudat et al., 2018 [33]			Switzerland	Primary care practice or ambulatory care clinic	>16 years attending with a complaint of chest pain (mean age of 55.2 years)	CVD, chest pain in ambulatory care	Referral to cardiologist at 12 months follow up	5 months	672	47.6:52.4
Leening et al., 2014 [34]			Netherlands	Part of the Rotterdam Study, a population-based study to determine the occurrence and determinants of age-related diseases in the general population	Community in Rotterdam aged ≥55 and free from CVD at baseline	CHD	First diagnosis of CHD, cerebrovascular disease, HF, cardiovascular disease, or death from non-cardiovascular causes.	3 years	8419	39.1:60.9
Leifheit-Limson et al., 2015 [35]			US and Spain	VIRGO (Variation in Recovery: Role of Gender on Outcomes of Young AMI Patients) study in US and Spanish hospitals	18 to 55 years old with AMI with a median age of 48 years	Acute MI	Prevalence of five cardiac risk factors	3 years and 5 months	3501	33:67
Mariani et al., 2013 [36]			Argentina	Epi-Cardio registry, Intensive Cardiovascular Care Units of Argentina (54 centres)	Patients diagnosed with acute coronary syndrome	ACS	Care in acute coronary syndrome	7 years	8997	72.4:28.6
Millett et al., 2018 [37]			UK	UK Biobank where participants attended one of 22 centres	Participants between 40 and 69 with a mean age of 56.2 years old	MI	Incidence of fatal or non-fatal MI	4 years	471,998	44:56
Mokhles et al., 2018 [38]			Netherlands	The national database of The Netherlands Association for Cardio-Thoracic Surgery	All patients >18 who underwent AV, or a combined CABG/AV surgery (mean age of females was 71 years and those of males 66 years)	CAD	Patient and procedural characteristics and early mortality after aortic valve and combined aortic valve/coronary	3 years and 11 months	14,584	60.5:39.5
Norgaard et al., 2015 [39]			Denmark	Data was obtained from the Western Denmark Heart Registry	Data from the Western Denmark Heart Registry, a clinical database within a population-based healthcare system regarding patients with chest discomfort and/or dyspnoea and low to intermediate pretest probability of CAD admitted for their first coronary CT angiography mean age around 60 years old	CAD	Coronary artery calcium score, coronary revascularisation	3 years	3541	46:54
Peters et al., 2018 [40]			United States	Data from Market Scan and Medicare databases (health insurance)	U.S. adults <65 years of age with commercial health insurance in the Market Scan database and U.S. adults ≥66 years of age with government health insurance through Medicare, who filled a statin prescription in the last 30 days after discharge with MI	MI	Prescription of high-intensity statins following MI	1 year and 6 months	88,256	44:56
Virani et al., 2015 [41]			United States	Veterans’ Health, primary care clinics (130 facilities)	Adult patients with CVD (CHD, PAD, and ischemic stroke) with a mean age between 66-71 years old	CHD, PAD, and ischemic stroke	Prescription of statin or non-statin lipid-lowering agent (bile acid-binding resin, niacin, and ezetimibe)	11 months	972,532	98.6:1.4
Worrall-Carter et al., 2016 [42]			Australia	Database maintained by Victorian state government that included data in Victorian hospitals, rehabilitation centres, extended care facilities and day procedure centres	All patients admitted to Victorian hospitals with a first-time diagnosis of ACS	ACS	Epidemiology, treatment, and outcomes of patients admitted with a primary diagnosis of ACS	2 years and 1 month	28,985	64:36
Zhao et al., 2017 [43]			Asia, Europe, and the Middle East	Outpatient cardiology clinics in 11 countries across three regions: Europe (Belgium, Croatia, Denmark, Ireland, Italy, Northern Ireland, Romania, and Russia); Asia (Taiwan and China); and the Middle East (Saudi Arabia).	Patients aged ≥18 years with established CHD (defined as a history of CABG, PCI, ACS, or stable angina) with a mean age of around 65 years old	CHD	Risk factors in patients with CHD	1 year	10,112	71:29
Median and interquartile range								4	10,112	47.6:52.4 (IQR, 44:56-32.5:67.5)
	Case-Control									
Daly et al., 2006 [44]			Europe	The Euro Heart Survey of Stable Angina	All patients with a clinical diagnosis of stable angina considered mean age 61 years old	Stable angina	Occurrence of death or myocardial infarction and the occurrence of all CV events*	1 year	3779	58:42
Hyun et al., 2017 [45]			Australia	60 Australian primary healthcare services	Adult patients above 35 years old	CHD, Ischaemic stroke or peripheral vascular disease.	CVD risk factors and recommended medications	9 months	13,294	53:47
Kislitsina et al., 2019 [46]			United States (Chicago)	Cardiovascular Research Database of the Clinical Trial Unit of the Bluhm Cardiovascular Institute at Northwestern Memorial Hospital	All patients who underwent mitral valve surgery mean age of 61.9 years	Mitral Valve Disease	Characterisation of post-operative hospitalisation event and analysis of early and late complications	13 years and 3 months	1436	58.2:41.8
Lee et al., 2019 [47]			Australia	General practice dataset in Australia (known as MedicineInsight) from 438 sites across Australia	≥18 years with a history of CHD who were considered active patients (≥ 3 encounters in MedicineInsight database over 2 years)	CHD	Recommended medication, cardiovascular risk factors, achieve treatment targets	4 years	130,926	52.3:46.7
Median and Interquartile range								4	8536.5	55.5:44.5 (IQR,53:47-41.95:58.05)
	Case Series									
Conti et al., 2002 [48]			Sao Paulo, Brazil	Instituto do Coração of the Hospital das Clínicas of the Medical School of the University of São Paulo	25- to 45-year-old patients with the diagnosis of acute myocardial infarction (mean age 41 years old)	Acute MI	Differences in risk factors and treatment between males and females with acute MI	3 years and 7 months	236	77:23
	Cross-Sectional									
Murga-Eizagaetxebarría et al., 2019 [49]			Spain	OFRECE study: primary care setting in Spain (425 consultation rooms in 46 provinces in Spain)	Spanish population aged 40 years and older that consulted for chest pains and/or palpitations	Chest pain and palpitation	Clinical management of patients with symptoms of chest pain and/or palpitations	-	1132	32:68
Rachamin et al., 2021 [51]			Switzerland	FIRE study: FIRE database that collected data from electronic medical records of > 500 general practices in Switzerland.	40- to 79-year-old patients with at least two consultations (one before 2017 and one during 2018).	CVDs	LDL-cholesterol, blood pressure and HBA1c	-	59,092	48.1:51.9
Xia et al., 2020 [50]			China	Door to door surveys in community in seven geographical regions in China (Northeast, North, Northwest, East, Central, South, and Southwest China). (4000 residents in 39 communities)	Adults ≥45 years old who have been living in their area for more than 6 months whether urban or rural of median age in the range of 59 – 62 years old	CVDs	CVD risk factors	2 years and six months	47,841	38.7: 61.3

The Joanne Briggs Institute (JBI) checklist was used to assess the quality of included case-control, case series, and cross-sectional studies [25]. In addition, the Critical Appraisal Skills Programme (CASP) checklist was used to assess the cohort studies [26]. Each article was subjected to evaluation by two independent reviewers (RB and AA). Any differences in ratings were resolved by consensus among reviewers. All studies were included after passing the evaluation criteria.

Data Analysis

Data were extracted using Statistical Product and Service Solutions (SPSS, v26; IBM SPSS Statistics for Windows, Armonk, NY). The summary statistics were reported for study duration, sample size, and men-to-women ratio. After conducting the test of normality, the data did not show normal distribution, so median and interquartile ranges (IQR) were evaluated. The differences in diagnosis, risk factors, and medicines prescribed for men and women patients with CVDs were also reported. As the included studies had high heterogeneity, the findings were presented as a narrative summary instead of a meta-analysis, using tables and figures to aid presentation where appropriate.

List of Definitions

CVDs represent a group of disorders that affect the heart and blood vessels [27]. ICD-11 codes of CVDs used included arrhythmia (BC60-BC65), hypertension (BA00-BA04), coronary artery disease (CAD) (BA80-BA86), heart failure (HF) (BD10-BD14), IHD (BA40-BA43), and mitral valve disease (MVD) (BB60-BB65) [28]. Diagnosis is the process of identifying a disease, condition, or injury from its signs and symptoms [29]. Implicit bias operates in an unintentional, unconscious manner, silently exerting its influence on perception, memory, and behaviour [30]. Prevention refers to actions taken to lower the risks of a disease occurring [27]. Biological sex is assigned at birth, depending on the appearance of the genitals. Gender identity is the gender that a person "identifies" with or feels themselves to be [27]. A risk factor is any attribute, characteristic, or exposure of an individual that increases the likelihood of developing a disease or injury [31]. Treatment means the management and care of a patient to combat disease or disorder [32].

Results

Study Extraction

Studies included were those focused on gender bias in the diagnosis, prevention, and/or treatment of CVDs. Studies evaluating gender in different contexts to the three aforementioned contexts were excluded. Moreover, studies having CVDs as secondary conditions or those that did not fulfill the ICD-11 criteria for the cardiovascular (CV) condition were excluded. Descriptive studies that had no focus on patients’ outcomes were also excluded.

Only peer-reviewed articles were considered; yet, no language restriction was applied. A total of 977,032 articles were screened through the six databases by two reviewers (RB and SA) independently, where 6,400 were obtained after initial screening and removal of duplicates (Figure 1). Out of the 6,400, 6,336 were excluded based on their title considering the inclusion/exclusion criteria. Of the remaining 64 studies, 31 studies were excluded after evaluation of the abstracts according to the inclusion/exclusion criteria. Of the remaining 33 full-text studies, 14 were found to be irrelevant. Consequently, the research resulted in 19 studies that were subject to quality assessment by two reviewers (AA and RB) who scored independently. All of the 19 studies scored high and were included in the review (Figure 1).

Study Characteristics

The characteristics of the included studies are listed in Table 1. Study publication dates ranged from 2002 to 2021. The study sample size varied between 236 and 972,532, with a median range of 8,767 participants (IQR 3,511-25,385). Out of the aforementioned studies, 11 studies were cohort studies [33-43], four case-control [44-47], and four case series [48-51]. Where specified, studies were conducted in 17 countries across the globe: Argentina [36], Australia [42,45,47], Belgium [43], Brazil [48], China [43,50], Croatia [43], Denmark [39], Ireland [43], Netherlands [34,38], Romania [43], Russia [43], Spain [35,49], Saudi Arabia [43], Switzerland [33,51], Taiwan [43], the UK [37], and the US [40,41,46]. However, one study only reported conducting a survey across the continent across multiple countries [44]. The study settings reported for the 19 studies were ambulatory care settings (n = 1), community (n = 3), databases (n = 5), hospitals (n = 4), and primary healthcare services (n = 7). Patients reported in the aforementioned studies were in the age range of 16-69 years old and had current or previous cardiac conditions. Where a current cardiac condition was reported, the specific CVD diagnosis comprised: acute coronary syndrome (ACS) (n = 2), CAD (n = 1), CHD (n = 5), IHD (n = 1), ischemic stroke (n = 1), MVD (n = 1), MI (n = 4), peripheral vascular disease (PVD) (n = 1), and stable angina (n = 1). The duration of the study ranged between one year and 13 years, with a median duration of four years (IQR 1.5-4). The median men-to-women ratios for cohort and case-control studies were 47.6:52.4 (IQR 44:56-32.5:67.5) and 55.5:44.5 (IQR, 53:47-41.95:58.05), respectively. In addition, the case series and cross-sectional studies had men-to-women ratios of 77:23 and 32:68, respectively.

Reported CVDs in Women Compared to Men

Reported CVDs differed between men and women that depended on gender, age, comorbidities, and lifestyle. In certain conditions related to atherosclerosis, women showed milder symptoms than men; yet, they were at higher risk of developing multiple comorbidities than men [42]. Moreover, in CVDs such as ACS, women were more likely to be admitted to hospital at older age, where old age represented an additional risk factor for ACS [52]. Women admitted at older age to hospital could further explain the higher rates of stroke and heart failure among women [34-36,40,46].

Nonetheless, the prevalence of ACS and its manifestations (ST-elevated myocardial infarction (STEMI), non-ST elevated myocardial infarction (NSTEMI), and unstable angina) has been reported higher among men in four studies [35,37,49]. For stable angina, the prevalence among genders varied between men and women depending on the severity when assessed by the Canadian Cardiovascular Society Grading Scale [53]. In this respect, women showed more class II and less class I symptoms in mild-to-moderate angina [44]. It is worth mentioning that class II symptoms were more severe than class I symptoms. Patients with class II symptoms experienced limitations in carrying out normal everyday activities such as walking or taking stairs [53].

Differences between women and men in the prevalence of ACS and stable angina in terms of severe outcomes in women indicated underdiagnosis of women’s symptoms. This was confirmed in additional studies that reported higher rates of PAD and chronic kidney disease (CKD) among women where both PAD and CKD represented modifiable risk factors for ACS [35,40,41,47]. Moreover, two of the aforementioned studies reported higher rates of CAD among women [35,47] contrary to the literature where men showed higher rates of CAD than women [34,40,41,46]. This could be related to CV symptoms being misdiagnosed among women as anxiety or gastrointestinal-related symptoms [39].

CV Risk Factors in Women Compared to Men

Women were less likely to have their CV risk factors assessed than men and that impacted CVD prevention among women [45,51]. Eight risk factors were mentioned in this sense: body mass index (BMI), hypertension (HTN), hypercholesterolaemia, hyperlipidaemia, hypertriglyceridemia, smoking, and family history of CVDs (Table 2).

**Table 2:** Prevalence of CVDs risk factors in men and women patients - = not reported. % refers to the percentage of male and then female participants separately. *Comparisons with p = <0.001 significant, **p = 0.002, ***p = 0.24, a p = 0.031 Murga-Eizagaetxebarria et al.'s study first set for chest pain and second for atrial fibrillation [44].

Study	Risk Factors													
	Smoking (%)		Diabetes (%)		Hypertension (%)		Hypercholesterolemia (%)		Hypertriglyceridemia (%)		Hyperlipidaemia (%)		Family history of CVD (%)	
	Men	Women	Men	Women	Men	Women	Men	Women	Men	Women	Men	Women	Men	Women
Daly et al., 2006 [44]	69.1*	30.1*	17.1	18.8	58.6*	66*	-	-	-	-	57.2***	59.3***	-	-
Hyun et al., 2017 [45]	22.9*	20*	45.7*	52.6*	18.6	20.7	4.5	4.9	-	-	-	-	-	-
Kislitsina et al., 2019 [46]	-	-	13	13	58	55	-	-	-	-	-	-	-	-
Lee et al., 2019 [47]	11.7*	9.9*	34.7*	29.6*	95.1*	93.5*	76.9*	69.7*	81.4*	75.8*	-	-	-	-
Conti et al., 2002 [48]	74.7	63	9.3	9.3	52.7	40.7	54.9	40.7	34.1	22.2	-	-	48.9	37
Clerc Liaudat et al., 2018 [33]	-	-	-	-	-	-	-	-	-	-	-	-	-	-
Leening et al., 2018 [34]	26.7*	20.5*	11.8	9.1	21	22	5.6*	6*	-	-	-	-	15.8	16.2
Leifheit-Limson et al., 2015 [35]	59.3	59.7	26.7*	38.8*	62.3	63.8	92.1*	82.6*	-	-	92.1	82.6	73.5	76
Mariani et al., 2013 [36]	36.3*	18.6*	19.2*	23.1*	59.2*	69.6*	45.1	44.7	-	-	45.1	44.7	-	-
Millett et al., 2018 [37]	12.4	8.8	5.7	3.3	49.6	44.1	-	-	-	-	-	-	-	-
Mokhles et al., 2018 [38]	-	-	-	-	5.7	6.4	-	-	-	-	-	-	-	-
Nørgaard et al., 2015 [39]	23.8*	19.4*	9.2**	6.4**	-	-	-	-	-	-	-	-	45.4*	53*
Peters et al., 2018 [40]	-	-	33.9	37.3	-	-	-	-	-	-	-	-	-	-
Virani et al., 2015 [41]	-	-	43*	38.9*	80	77.7	42	51.3	-	-	-	-	-	-
Worrall-Carter et al., 2016 [42]	49*	27*	-	-	64*	69*	-	-	-	-	-	-	-	-
Zhao et al., 2017 [43]	18.7	9.5	31.9	40.3	71.9	80.8	67.8	67.1	-	-	67.8	67.1	31.1	33.3
Murga-Eizagaetxebarría et al., 2019 [49]	19.8* 21	12.4* 18.8	18.9 16	14.6 11.1	60.9 60.6	59.7 49.3	47.5* 34.7	35.9* 26.3	-	-	-	-	23.3 23.7	25.6 24.4
Xia et al., 2020 [50]	37.1	6.4	19	19.1	54.8	51.7	-	-	-	-	31.3	34.2	-	-

BMI was reported significantly higher in women than men in four studies [34,35,43,49], significantly lower in women than men in two studies [39,47], and not significantly different between both genders in two studies [37,46]. Similarly, diabetes was reported significantly higher in women than men in five studies [35,36,40,43,44], significantly lower in women than men in six studies [37,39,45,47-49], and not significantly different between both genders in two studies [46,50].

HTN was reported significantly higher in women than men across eight studies [35,36,38,42-45,47] and significantly lower in women than men in seven studies [37,39,41,46,48-50].

Hypercholesterolaemia was reported significantly higher in women than men across five studies [34,41,43,45,51] and significantly lower in four other studies [45,47-49]. Higher rates for hyperlipidaemia were stated for men than women across four studies [35,36,43,50] and higher rates in women than men in three studies [41,44,51]. Hypertriglyceridemia was reported only in three studies that showed significantly higher rates in men than women [47,48]. Smoking was consistently higher in men across all the studies that reported it [34-37,39,43-50,52]. Family history of CVDs was reported higher in women than men across six studies [34,35,39,43,49] and lower in one study [48].

Assessment and Management of CVDs in Women

Women had less assessment of essential and complementary examination of their medical condition in chest pain, angina, and CHD. When complaining of chest pain, women received an equal assessment of the essential examination (i.e., chest pain severity) but were offered less complimentary examination and were referred less to cardiologists or hospitalisation [33,49]. On the other hand, men received more complementary examinations, including coronary angiography, ergometry, ECG, and cardiac enzymes (creatinine kinase and troponin) [33,49]. Similarly, in the initial assessment of angina, exercise ECG and coronary angiography were performed less for women [44].

In CHD patients, women were 2.5 times less likely to be referred to a cardiologist than men and that indicated stronger gender bias in the management of the condition [33]. In the latter study, referral of women to cardiologists was less encountered when male physicians undertook the initial diagnosis rather than women physicians. Subsequently, women overall were less likely to achieve treatment targets in CHD [43].

Management of CVDs was performed less in women whether admitted to hospital with ACS post-diagnosis of MI. After admission to a hospital with ACS, women were less likely to receive coronary intervention regardless of the type of ACS [52]. In addition, in-hospital mortality following concomitant coronary artery bypass graft (CABG) was significantly higher in women than in men [38]. Furthermore, post-diagnosis of MI, women had longer waiting times to get treatment and undertook less frequent chemical/mechanical thrombolysis than men [48]. Moreover, invasive coronary angiography and revascularisation after follow-up post-CAD were performed significantly less in women than in men [39,44,49]. This could be partly due to significantly lower levels of coronary artery calcium in women [49].

Prescription of Medicines to Women

Six medicines/medicine classes were reported: antihypertensive, antiplatelets (including aspirin), beta-blockers and lipid-lowering agents (including statins), oral anticoagulants, and unspecified antianginal drugs (Table 3). It is noteworthy to mention that aspirin was often reported separately from the remaining antiplatelets, and statins were reported separately in studies from the remaining lipid-lowering agents.

**Table 3:** Medications prescribed to men and women CVD patients - = not reported. % refers to the percentage of male and then female participants separately. *significant male-to-female difference p = <0.001 Specified antihypertensive medicines in Lee et al.'s [47] study include short-acting nitrates, angiotensin-converting enzyme inhibitors (ACEIs), and angiotensin receptor blockers (ARB). Specified antihypertensive medicines in Mariani et al.'s [36] study include ARB, ACEIs, and calcium channel blockers (CCB). Millett et al. [37] reported antianginal: nitrate (in men 9.8 and women 12.4%). The study by Verani et al. [41] reported statins and high-intensity statins. Antihypertensive medicines reported by Zhao et al. [43] include total antihypertensive, ACE, ARB, and CCB. Zhao et al. [43] reported antianginal nitrates (30.5% in men and 37% in women). Statins reported in Rachamin et al.'s [51] study include low intensity, medium intensity, and high intensity (as primary and secondary prevention of CVDs); the antihypertensive medicines reported were ARB and CCB as primary and secondary prevention of CVDs, respectively.

Study															Prescribed Medication(s)						
	Antiplatelet (%)		Aspirin (%)			Lipid-lowering agents (%)				Statin (%)				β-Blocker (%)				Oral anticoagulant		Antihypertensive	
	Men	Women	Men		Women	Men		Women	Men			Women	Men			Women	Men		Women	Men	Women
Daly et al., 2006 [44]	84*	76*	81*		73*	53*		47*	51*			45*	67			65	-		-	-	-
Hyun et al., 2017 [45]	57.4*	53.4*	7.9		7.6	69.1		67.5	69.1			67.5	78.1*			80.2*	7.9		7.6	78.1	80.2
Lee et al., 2019 [47]	46.02*	32.08*	46.02*		32.08*	-		-	79.4*			61.2*	52.3*			41.1*	-		-	34.8	30.1
Leening et al., 2018 [34]	-	-	-		-	-		-	8.2			9.9	22			26.5	-		-	22	26.5
Mariani et al., 2013 [36]	71.9	64.6	95.1		94.3	-		-	89.6*			84.7*	86.8*			81.2*	-		-	9.8	12.4
Millett et al., 2018 [37]	-	-	-		-	14.2		8.8	-			-	-			-	-		-	15.6	12.3
Nørgaard et al., 2015 [39]	-	-	-		-	35.9		32.3	-			-	-			-	-		-	37.7	40
Peters et al., 2018 [40]	61.7*	54.5*	-		-	10.7*		9.2*	14*			12*	81.2			79.5	-		-	-	-
Rachamin et al., 2021 [51]	-	-	-		-	-		-	1/1.7			1.3/3.4	18.9/61.3			19.9/56.8	-		-	50.5/12.7	54.4/12.3
Virani et al., 2015 [41]	-	-	-		-	-		-	64.8			57.6	-			-	-		-	-	-
Zhao et al., 2017 [43]	91.7	86.3	92.2		92.1	-		-	83.5			75.7	73			68.4	-		-	92.2	92.1

Women were less likely to be prescribed CV medications than men in the same age group [43,45]. However, that was not the case for antianginal nitrates and antihypertensive treatment. Antianginal nitrates were more significantly prescribed in women than men in two studies [36,43]. Similarly, antihypertensive medicines were more prescribed in women than men in most studies and could be related to the higher prevalence of HTN among women [34,36,39,45,51]. Not all studies reported the specific class of antihypertensive reported, but where reported, angiotensin-converting enzyme inhibitors [36,43,47], angiotensin II receptor blockers [43,47,51] and calcium channel blockers have been used [36,43,51]. Where reported, beta-blockers were more prescribed in women than men in three studies [34,45,51] and less prescribed in five studies [36,40,43,44,47].

For antiplatelets (including aspirin), women were less often prescribed antiplatelets than men after initial assessment of angina and hence were more likely to suffer MI or death in the one-year follow-up after angina [44,45].

Lipid-lowering agents (including statins) were more significantly prescribed in men [41,44,45,51] despite that women showed high levels of low-density lipoprotein [51]. Following hospital discharge of MI, women were prescribed significantly less high-intensity statins (atorvastatin and rosuvastatin) than men, which was not related to differences in sociodemographic factors, comorbidities, and healthcare utilisation [40].

Women Patients’ Awareness Towards Preventive Measures

Only two studies indicated women patients’ awareness towards CVDs [35,49]. The aforementioned two studies found that men patients were more likely to consider themselves at risk of heart disease and consult with a primary care physician and/or a cardiologist than women patients. On the contrary, women patients did not always consider their risk factors as related to CVDs nor were told by healthcare professionals that they were at risk and/or how to minimise the risk [35].

Discussion

The findings of this review suggested the presence of gender bias against women in relation to CVDs’ diagnosis, prevention, and treatment. The studies included in the review mainly reported under consideration of gender-specific requirements. Hence, studies either did not consider CV risk factors, diagnostic tests, and/or medicines among women or considered them and found them more tailored to men. Therefore, this review highlighted gender bias encountered in the diagnosis, prevention, and treatment of CVDs among women. Two previous systematic reviews assessed the risks of CVDs in women patients; however, they did not address the diagnosis and/or treatment of CVDs [52,54]. Similarly, two other reviews were narrative in nature and only assessed the gender bias specific to IHD in women [16,17]. Consequently, by including further diagnosis and treatment, this review added to the previous literature and provided useful insight for both patients and healthcare professionals working with CVDs. It is noteworthy to mention that the review did not show selection bias in the women-to-men ratios in the individual studies that showed almost equal ratios with a median of 53:47 (IQR 44:36-64:56).

With regards to CVD risk factors, HTN was most reported in women, while men showed higher rates of smoking [39,47,55]. Women only started heavy smoking in the US and the UK in the 1940s [55]. Smoking increases the risk of atherosclerosis and its manifestations (IHD and PAD) [56]. Hence, over-considering the aforementioned risk factors that are more prevalent in men could lead to unconscious misdiagnosis of CVDs among women.

In relation to CVD diagnosis, women received fewer diagnostic tests than men, which could be explained by the differences in sensitivity towards the tests between both genders. This could be explained by the different manifestations of CVD in women when compared to men [17,57,58]. Women show a delayed onset of CVDs than men, and women’s symptoms are often described as atypical, leading to underappreciating the severity of CVDs [33]. A review by Canto et al. demonstrated that women with ACS are more likely to present with no chest discomfort than men and that is attributed to the underdiagnosis of CVDs in women [59]. Men are known to have larger more visible blockages in coronarys compared with women [16]. This could further explain why women experience worse clinical outcomes than men [17,22,60], with the disease not being diagnosed and treated as efficiently as it could be. In other studies, physicians misdiagnosed CHD symptoms in women as gastrointestinal or mental health conditions’ symptoms [61,62]. Physicians-patient interaction has been shown to differ when interacting with men versus women patients where three main factors play a role, including physician’s prejudices, symptom perception, and gender-specific description [63]. Though guidelines exist regarding differences in genders [8-13], physicians may not be aware of them or may think that CVD is a man’s disease [64,65]. Hence, gender stereotyping and bias represent potential reasons for gender disparities in the diagnosis of CVD [11,66,67]. These findings suggest the need for more intensive investigation for women patients [16,17].

This bias not only affected diagnostic tests but also other procedures relating to the assessment and management of CVDs. For instance, men received more revascularisation than women despite their unwillingness to undertake invasive procedures [68]. Hence, men were seven times more likely than women to refuse invasive investigation [69].

Furthermore, women received fewer prescribed medicines when compared to men. Men were reported to have better access to four out of six key medicines/classes (antianginal, antiplatelets, oral anticoagulants, and statins) in comparison to women [17,44,60]. This was consistent with studies in the literature that reported women were more likely to receive fewer medicines for CVDs than men [60,70]. Even when prescribed statins, women are more likely to be prescribed lower doses of statins than men [41]. This was encountered more with high-intensity statins despite that the American College of Cardiology/American Heart Association (ACC/AHA) cholesterol guideline did not make a differentiation by gender in relation to statin prescription [12].

Patient awareness of CVDs was underreported in the included studies. However, it was stated that women had considered themselves at less risk of CVDs and were less likely to visit a primary care physician [71. However, men considered their symptoms more seriously and were more likely to visit a cardiologist [71]. In similar reviews, women were found to be more likely to call 911 for a friend experiencing heart attack symptoms than for themselves and less likely to report their own symptoms [7,21,72-75]. The latter finding was confirmed by a survey that interviewed 1,000 American women who had reported their lack of awareness of CVDs being the main cause of death among women [74]. The lack of awareness could be partly caused by stress and partly socioeconomic factors where more women live in poverty than men and that contributes to higher rates of anxiety that is strongly linked to adverse CV events [75]. Future work to improve this awareness is necessary to ensure women patients have access to healthcare before conditions worsen.

There were limitations in this review. Although rigorous and systematic, the reviewers did not include unindexed and unpublished research. Furthermore, it had strict selection criteria that affected the number of studies yielded and generalisability of the findings. Thus, the review had a low number of studies (n = 19), and that was related to the definitions and the inclusion criteria used to increase sensitivity. Moreover, there was an imbalance in the CVD evaluated in the studies. Thus, only three studies evaluated CVDs in general, and the remaining studies explored ACS, CAD, CHD, MI, MVD, or PVD. This introduced heterogeneity in the studies. Hence, the included studies were of different findings and variable quality with considerable heterogeneity between the included studies particularly with variation in reporting statistics. Owing to the significant heterogeneity, a meta-analysis was not conducted. As the spread of the studies occurred over the last 20 years, this affected the generalisability of the findings. Furthermore, no clear definition of gender bias was used by any of the included studies with inconsistencies in the term used. There was no way of assessing the severity of the CV conditions reported and that affected the outcomes. In addition, the review was limited to quantitative studies, which could be enhanced by the addition of qualitative studies that may further uncover barriers to women’s access to CV diagnosis and therapy. However, this was limited considering the low availability of the published literature regarding women’s perspectives regarding CVDs. Nonetheless, the studies in this review included a significant number of women sufficient to provide evidence of women’s experience with CVDs.

## Conclusions

The findings of this review have emphasised gender bias associated with the prevention, diagnosis, and treatment of CVDs in women. The review identified poor access to diagnostic tests and CV medicines in women in comparison to that in men. These findings suggest the need to consider further gender-specific requirements in applying clinical guidelines for the management of CVDs. Future research should also consider physiological differences between men and women in the diagnosis and treatment of CVDs.

## Appendices

Supplementary file S1

**Table 4:** PRISMA checklist From: Moher et al. [23] For more information, visit www.prisma-statement.org.

Section/topic	#	Checklist item	Reported on page #
TITLE			
Title	1	Identify the report as a systematic review, meta-analysis, or both.	1
ABSTRACT			
Structured summary	2	Provide a structured summary including, as applicable: background; objectives; data sources; study eligibility criteria, participants, and interventions; study appraisal and synthesis methods; results; limitations; conclusions and implications of key findings; systematic review registration number.	1,2
INTRODUCTION			
Rationale	3	Describe the rationale for the review in the context of what is already known.	4,5
Objectives	4	Provide an explicit statement of questions being addressed with reference to participants, interventions, comparisons, outcomes, and study design (PICOS).	5
METHODS			
Protocol and registration	5	Indicate if a review protocol exists, if and where it can be accessed (e.g., Web address), and, if available, provide registration information including registration number.	NA
Eligibility criteria	6	Specify study characteristics (e.g., PICOS, length of follow-up) and report characteristics (e.g., years considered, language, publication status) used as criteria for eligibility, giving rationale.	6
Information sources	7	Describe all information sources (e.g., databases with dates of coverage, contact with study authors to identify additional studies) in the search and date last searched.	5,6
Search	8	Present full electronic search strategy for at least one database, including any limits used, such that it could be repeated.	5,6
Study selection	9	State the process for selecting studies (i.e., screening, eligibility, included in systematic review, and, if applicable, included in the meta-analysis).	5,6
Data collection process	10	Describe method of data extraction from reports (e.g., piloted forms, independently, in duplicate) and any processes for obtaining and confirming data from investigators.	6,7
Data items	11	List and define all variables for which data were sought (e.g., PICOS, funding sources) and any assumptions and simplifications made.	7,8
Risk of bias in individual studies	12	Describe methods used for assessing risk of bias of individual studies (including specification of whether this was done at the study or outcome level), and how this information is to be used in any data synthesis.	6,7
Summary measures	13	State the principal summary measures (e.g., risk ratio, difference in means).	7
Synthesis of results	14	Describe the methods of handling data and combining results of studies, if done, including measures of consistency (e.g., I^2^) for each meta-analysis.	8-10

Supplementary file S2

**Table 5:** Medline search strategy Database: Medline Ovid (2000-2022)

Term	Synonym
Cardiovascular disease*	Cardiovascular disorders or heart diseases or heart conditions or cardiovascular defects
Access	Availability or accessibility
Female*/	Women or gender or sex
Prevention/	Avoidance
Treatment/	Therapy or medication
